# Nitric oxide-dependent micro- and macrovascular dysfunction occurs early in adolescents with type 1 diabetes

**DOI:** 10.1152/ajpendo.00267.2021

**Published:** 2021-12-13

**Authors:** Linda A. Jahn, Brent Logan, Kaitlin M. Love, William B. Horton, Natalie Z. Eichner, Lee M. Hartline, Arthur L. Weltman, Eugene J. Barrett

**Affiliations:** ^1^Department of Medicine, School of Medicine, College of Arts and Sciences, University of Virginia, Charlottesville, Virginia; ^2^Department of Pediatrics, School of Medicine, College of Arts and Sciences, University of Virginia, Charlottesville, Virginia; ^3^Department of Kinesiology, School of Medicine, College of Arts and Sciences, University of Virginia, Charlottesville, Virginia; ^4^Department of Pharmacology, School of Medicine, College of Arts and Sciences, University of Virginia, Charlottesville, Virginia

**Keywords:** endothelial dysfunction, microvascular function

## Abstract

Arterial stiffness and endothelial dysfunction are both reported in children with type 1 diabetes (DM1) and may predict future cardiovascular events. In health, nitric oxide (NO) relaxes arteries and increases microvascular perfusion. The relationships between NO-dependent macro- and microvascular functional responses and arterial stiffness have not been studied in adolescents with DM1. Here, we assessed macro- and microvascular function in DM1 adolescents and age-matched controls at baseline and during an oral glucose challenge (OGTT). DM1 adolescents (*n* = 16) and controls (*n* = 14) were studied before and during an OGTT. At baseline, we measured: *1*) large artery stiffness using both aortic augmentation index (AI) and carotid-femoral pulse wave velocity (cfPWV); *2*) brachial flow-mediated dilation (FMD) and forearm endothelial function using postischemic flow velocity (PIFV); and *3*) forearm muscle microvascular blood volume (MBV) using contrast-enhanced ultrasound. Following OGTT, AI, cfPWV, and MBV were reassessed at 60 min and MBV again at 120 min. Within individual and between-group, comparisons were made by paired and unpaired *t* tests or repeated measures ANOVA. Baseline FMD was lower (*P* = 0.02) in DM1. PWV at 0 and 60 min did not differ between groups. Baseline AI did not differ between groups but declined with OGTT only in controls (*P* = 0.02) and was lower than DM1 at 60 min (*P* < 0.03). Baseline MBV was comparable in DM1 and control groups, but declined in DM1 at 120 min (*P* = 0.01) and was lower than the control group (*P* < 0.03). There was an inverse correlation between plasma glucose and MBV at 120 min (*r* = –0.523, *P* < 0.01). No differences were noted between groups for V̇O_2max_ (mL/min/kg), body fat (%), or body mass index (BMI). NO-dependent macro- and microvascular function, including FMD and AI, and microvascular perfusion, respectively, are impaired early in the course of DM1, precede increases of arterial stiffness, and may provide an early indicator of vascular risk.

**NEW & NOTEWORTHY** This is the first study to show that type 1 diabetes impairs multiple nitric oxide-dependent vascular functions.

## Graphical abstract

**Figure:**
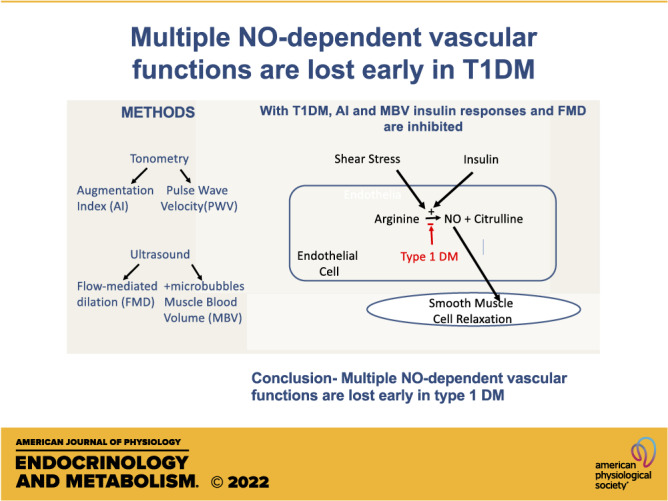

## INTRODUCTION

Cardiovascular disease (CVD) is the leading cause of death in people with type 1 diabetes (DM1) (1). CVD mortality rates among adults with DM1 exceed those of the general population by approximately two- to threefold even with excellent glycemic control (2), and women experience twice the excess risk of fatal and nonfatal vascular events compared with men with type 1 diabetes. Microvascular disease contributes to this via effects on the kidneys, myocardium, and other tissues. Microvascular complications are rare during the first 8–10 yr after DM1 diagnosis (3); however, increased arterial stiffness (4, 5), endothelial dysfunction in conduit vessels (6, 7), and intima-media thickening within the carotid artery (8, 9) have each been reported in children with DM1, suggesting early vascular injury in larger vessels. Central aortic vascular stiffness and endothelial dysfunction are discrete measures of vessel health and independently predict CVD events (10–12). Considering microvascular function, Khan et al. (13) observed altered endothelium-dependent and independent changes in skin microvascular vasodilatory function in DM1 children using laser-Doppler flowmetry.

Hyperglycemia per se is a significant risk factor for micro- and macrovascular dysfunction. Chronic hyperglycemia has clearly been shown to injure the microvasculature over time (3, 14). On the other end of the spectrum, hypoglycemia acutely impairs nitric oxide (NO)-dependent endothelial function in healthy humans (15). In individuals with DM1, acute hypoglycemia and hyperglycemia both result in impaired flow-mediated dilation (FMD), increased reactive oxygen species production, and upregulated inflammatory markers (16).

Physical inactivity is also strongly linked to vascular dysfunction. For example, increased sedentary behavior is associated with both macrovascular (17) and microvascular dysfunction (18). Increased levels of exercise and fitness overall have also been shown to improve vascular function in children with DM1 (19) and are associated with improved all-cause mortality in adults with DM1 (20).

To our knowledge, no studies have measured both micro- and macrovascular function in DM1 adolescents to examine early stages of small and large vessel dysfunction. Here, we comprehensively studied vascular function in both adolescents with DM1 and age-matched healthy controls. Subjects were studied in the postabsorptive state and in response to an oral glucose tolerance test (OGTT) to test vascular responses to acute elevations in both insulin and glucose. We measured carotid-femoral pulse wave velocity (cfPWV) (an index of central aortic stiffness) and aortic augmentation index (AI) by applanation tonometry (21). We assessed flow-mediated dilation (FMD) (large artery endothelial function) and postischemic flow velocity (PIFV) to assess endothelial function in smaller resistance arterioles. To test microvascular responsiveness, we measured forearm skeletal muscle microvascular blood volume (MBV) using contrast enhanced ultrasound (CEU) (22, 23). We hypothesized that functional differences exist at multiple levels of the arterial tree between adolescents with DM1 and healthy controls. Prespecified hypotheses were that individuals affected by DM1 would have higher cfPWV and AI and lower baseline FMD, along with reduced insulin-mediated decreases in AI and cfPWV and reduced insulin-mediated increases in microvascular perfusion, indicating insulin resistance at the large arteries and microvasculature, respectively. Furthermore, we hypothesized that higher levels of cardiorespiratory fitness would be associated with less vascular dysfunction in youth with DM1.

## RESEARCH DESIGN AND METHODS

### Subject Characteristics

Based on inclusion and exclusion criteria, individuals with DM1 had hemoglobin A1c < 11%, no hyperlipidemia, were nonsmokers, not taking any medications or supplements known to affect vascular function (e.g., angiotensin-converting enzyme inhibitors, angiotensin, or β-adrenergic receptor blockers, fish oil, vitamins E or C, or aspirin) and had diabetes for at least 1 yr. Control subjects were healthy, had BMI < 26 kg/m^2^, were nonsmokers, not taking any medications or supplements known to affect vascular function, and had no first-degree relatives with either type 1 or type 2 diabetes. This study protocol was approved by the University of Virginia Institutional Review Board. All studies were performed within the University of Virginia Clinical Research Unit (CRU).

### Screening Visit

Subjects reported to the CRU after a 10-h overnight fast. Written informed consent was obtained from all parents and informed assent was obtained from all children. The visit included a history and physical exam, height, weight, and vital signs. Blood samples were obtained for complete blood count, lipid profile, comprehensive metabolic panel, C-peptide, HbA1c, and a serum pregnancy test for postmenarchal females. Urine was obtained for albumin/creatinine ratio.

### Measures of Fitness and Body Composition

On a second outpatient visit, maximal oxygen consumption (V̇O_2max_) was determined using a treadmill protocol with open circuit spirometry (Carefusion, Vmax CART, Yorba Linda, CA). A modified Balke walking protocol was used with an initial velocity of 80.5 m/min and a zero percent grade. After 2 min, the speed was increased to 120.25 m/min and subsequently the grade was increased by 2.5% every 2 min (speed was unchanged) until the subject reached volitional exhaustion. V̇O_2max_ was chosen as the highest 1 min V̇O_2_ attained during the test. Heart rate (HR) and blood pressure (BP) were obtained at rest and HR was continuously monitored using a 12-lead electrocardiogram (EKG). Borg Rating of Perceived Exertion (RPE) (24) was also reported at the end of each 2-min stage. Body density was measured by air displacement plethysmography BodPod (Life Measurement Instruments, Concord, CA).

### Day of Admission

**Figure 1.**
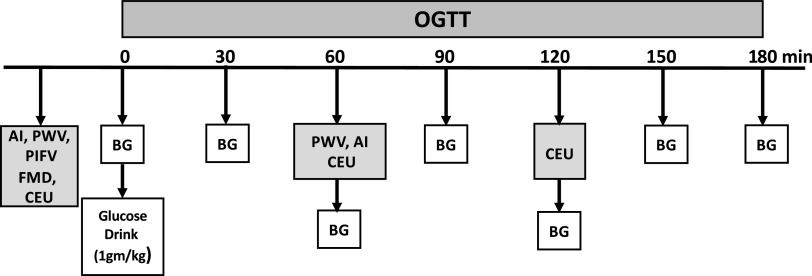
The time sequence of measurements obtained from each subject on the morning the oral glucose tolerance test (OGTT) was performed. Blood glucose (BG) measurements were obtained at baseline and every 30 min for 3 h after glucose ingestion. Vascular measurements are obtained at the times indicated. AI, augmentation index; CEU, contrast enhanced ultrasound; FMD, brachial flow-mediated dilation; PWV, pulse wave velocity; PIFV, postischemic flow velocity.

Approximately one week after measures of fitness and body composition were obtained, subjects reported to the CRU. They were asked to refrain from alcohol, caffeine, and exercise 24 h before admission. All subjects arrived after a minimum 10-h overnight fast and subjects with DM1 were instructed not to take rapid or short-acting insulin that morning. A urine pregnancy test was performed in postmenarchal females. Subjects with DM1 had two IV catheters placed in the same arm: the first for sampling blood glucose levels as well as infusion of Definity microbubbles, and the second for intravenous insulin infusion to maintain blood glucose between 100 and 150 mg/dL before the OGTT. Those adolescents without DM1 had only one intravenous placed for blood sampling and infusion of Definity microbubbles. After resting for 1 h, baseline vascular measures were obtained. AI, cfPWV, FMD, PIFV, and CEU during the fasted state. Upon completion of these measurements, an oral glucose tolerance test (OGTT: 1 g/kg) was administered. For those subjects with DM1, regular (U-100) insulin was administered subcutaneously 20 min before consuming the glucose drink based on their usual insulin/carbohydrate treatment plan. AI and cfPWV were repeated at 60 min while CEU was performed at 60- and 120- min after oral glucose ingestion (Fig. 1).

### Measures of Conduit and Resistance Arterial Function

AI and PWV were assessed by applanation tonometry using the SphygmoCor system (AtCor Medical, Itasca, IL) (21). We measured AI at the radial artery and corrected to a heart rate of 75 beats/min using the manufacturer’s software. PWV was measured between the carotid and femoral arteries using the SphygmoCor system, as previously described (23). FMD and PIFV were determined using a SONOS 7500 ultrasound system (Philips Medical Systems, Andover, MA). The brachial artery was imaged ∼5 cm proximal to the antecubital fossa using B-mode ultrasound. Baseline measurements of brachial artery diameter and blood velocity were followed by the placement of a blood pressure cuff around the forearm and inflated to 250 mmHg for 5 min. Upon cuff deflation, blood velocity was obtained during the first 20 s and diameter obtained every 10 s from 30 to 120 s. Analysis for vessel diameter was conducted offline using Brachial Analyzer (Medical Imaging Applications, LLC, Coralville, IA).

### Measurement of Skeletal Muscle MBV

CEU was performed as described previously (22) using a SONOS 7500 ultrasound system (Philips Medical Systems, Andover, MA) with harmonic imaging during the continuous systemic infusion of Definity (Lantheus Medical Imaging, North Billerica, MA), a suspension of perflutren gas-filled lipid microbubbles. Once the microbubble concentration reached steady state in the circulation (∼4 min), several images were obtained at a pulsing interval (PI) of 1 cardiac cycle followed by an immediate increase to a PI of 20 cardiac cycles for 5 min (∼15 images). The average video intensity (VI) of the one cycle images was subtracted from the average VI of the 20 cycle images. The resulting VI provides a measure of the steady-state MBV. Full microbubble replenishment curves were not acquired in these studies to minimize total ultrasound energy exposure to these adolescents. These images were recorded from the forearm in the transaxial plane 5 cm distal to the antecubital fossa using an S3 phased array transducer and a pulse mechanical index of 1.5. Data were recorded digitally and analyzed off line using Q-laboratory software (Phillips Medical Systems, Andover, MA).

### Biochemical Analyses

Comprehensive metabolic panel, HbA1c, complete blood count, lipid profile, C-peptide, serum folate, serum pregnancy test, and urine for microalbumin were assayed at the University of Virginia Clinical Chemistry Laboratory. Plasma glucose was measured using a YSI glucose analyzer (Yellow Spring Instruments; Yellow Springs, OH).

### Statistical Analyses

Statistical analyses were conducted using the software package Sigma Plot (v 12, Systat Software, Inc; San Jose, CA). Results are presented as means ± standard error of mean (SEM). Independent *t* tests were employed to determine mean differences in fitness, body composition, AI, cfPWV, FMD, PIFV, fasting blood glucose, and HbA1C. A two-way repeated-measures ANOVA was used to assess differences in the MBV between control and DM1 groups at 0, 60, and 120 min. Glucose AUC during the OGTT (0, 30, 60, 90, 120, 150, and 180 min) was calculated for both groups. A *P* value of ≤0.05 was accepted as statistically significant.

## RESULTS

### Subject Characteristics

Table 1 presents the baseline clinical characteristics of the 16 DM1 (10 M/6F; aged 14 ± 0.4 yr) and 14 age-matched healthy controls (7 M/7F; aged 14 ± 0.5 yr). Participants with DM1 and controls were similar in terms of V̇O_2max_ (37.8 ± 2.0 vs. 40.2 ± 1.8 mL/kg/min), BMI (21.2 ± 0.8 vs. 19.5 ± 1.2, kg/m^2^), and percent body fat (19.3 ± 2.0 vs. 18.5 ± 1.8%). As expected, HbA1c (8.7 ± 0.3 vs. 5.3 ± 0.1%, *P* < 0.001) and fasting plasma glucose (136 ± 11 vs. 89 ± 1 mg/dL, *P* < 0.001) were significantly higher in the DM1 group. Systolic blood pressure was also higher in the DM1 group (111 ± 4 vs. 100 ± 3 mmHg, *P* = 0.04).

**Table 1.** Subject characteristics

	Control Group (*n* = 14)	DM1 Group (*n* = 16)	*P* Value
Sex	F(7) M(7)	F(6) M(10)	
Age, yr	14 ± 0.4	14 ± 0.5	0.85
Height, cm	167 ± 2.4	170 ± 3.0	0.43
Weight, kg	57 ± 2.3	60 ± 3.1	0.18
BMI, kg/m^2^	19.5 ± 1.2	21.1 ± 0.8	0.22
Body fat, %	18.5 ± 1.8	19.3 ± 2.0	0.75
Systolic BP, mmHg	100 ± 2.7	111 ± 4.0	0.04
Diastolic BP, mmHg	61 ± 1.8	66 ± 20	0.16
V̇O_2max_, mL/kg/min	40.2 ± 1.8	37.9 ± 2.0	0.38
Duration DM1, yr	N/A	6.8 ± 1.1	
Fasting blood glucose, mg/dL	89 ± 1.4	136 ± 11.2	<0.001
Hemoglobin A1c, %	5.3 ± 0.09	8.7 ± 0.3	<0.001

BMI, body mass index; DM1, type 1 diabetes.

### Artery Stiffness: cfPWV and AI

**Figure 2.**
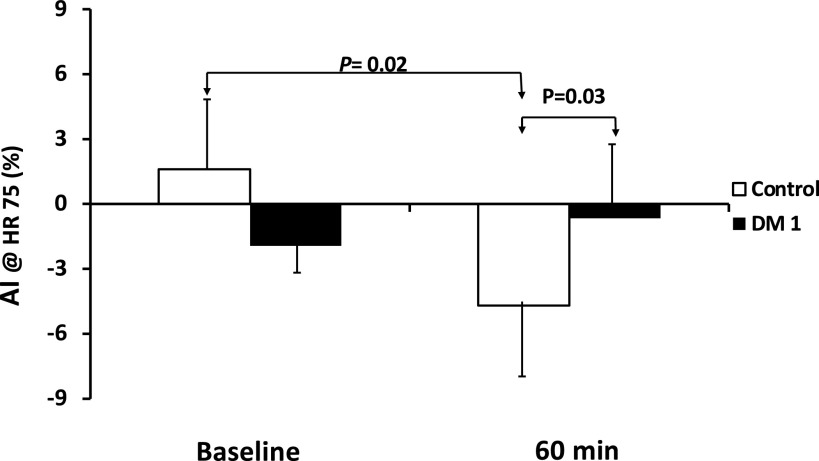
The values obtained for the augmentation index in the control and the type 1 diabetes patients at baseline and at 60 min after glucose ingestion. *P* values indicate results of unpaired *t* tests, for comparisons across, and results of paired *t* tests for within group comparisons. AI, augmentation index.

There were no baseline differences in cfPWV (5.3 ± 0.2 vs. 4.8 ± 0.2 m/s) or AI (−1.9 ± 3 vs. 1.6 ± 3%) between groups (DM1 vs. control). The cfPWV did not change after glucose challenge and no difference between groups was noted in cfPWV (5.2 ± 0.3 vs. 4.8 ± 2 m/s) at 60 min. However, AI trended higher in the DM1 group (0.7 ± 4%) and decreased significantly in controls (−4.7 ± 3%, *P* < 0.02), resulting in a significantly lower AI in the control group (*P* = 0.03) when compared with DM1 group at 60 min (Fig. 2).

### NO-Dependent Endothelial function: PIFV and FMD

There was no difference in baseline (87 ± 4 vs. 84 ± 4, cm/s) or peak flow velocity (145 ± 7 vs. 159 ± 10, cm/s) between the DM1 and control groups, respectively. However, the increase in PIFV trended higher in the control subjects 75 ± 72 vs. 49 ± 11 cm/s, *P* = 0.06. At baseline, FMD was significantly lower in the DM1 group (7 ± 1 vs. 11 ± 2%, *P* < 0.02), with 11 of 16 (69%) participants with DM1 having evidence of endothelial dysfunction based on established criteria of FMD ≤ 8% (11, 25).

### Microvascular Perfusion: MBV

**Figure 3.**
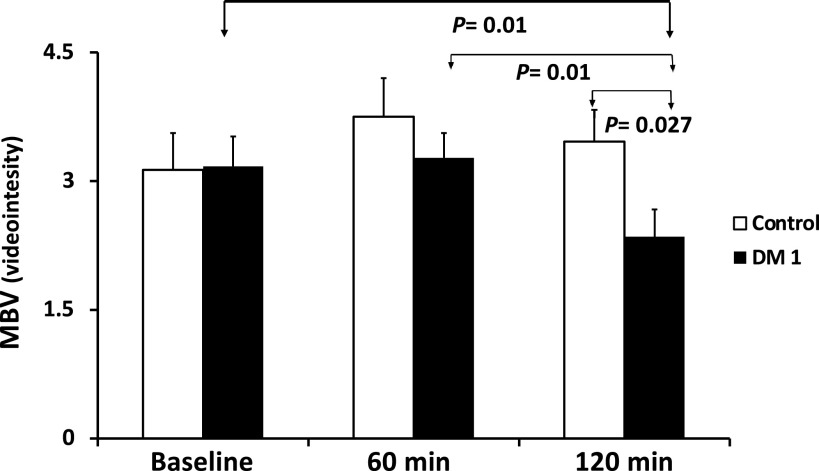
The microvascular blood volume (MBV) in arbitrary units of video intensity obtained at baseline and at 60 and 120 min after glucose ingestion. *P* values indicate results of unpaired *t* tests, for comparisons across, and results of paired *t* tests for within group comparisons.

There was no difference between DM1 and control groups at baseline [3.2 ± 0.4 vs. 3.1 ± 0.4 videointensity (VI) units] and 60 min (3.3 ± 0.3 vs. 3.8 ± 0.5 VI) (Fig. 3) for MBV. However, MBV declined significantly from baseline and 60 min to 120 min in participants with DM1 (*P* < 0.01 for each). MBV for the DM1 group at 120 min was also significantly lower than controls (2.4 ± 0.3 vs. 3.5 ± 0.4 VI, *P* < 0.03).

### Glucose and Insulin Responses to OGTT

**Figure 4.**
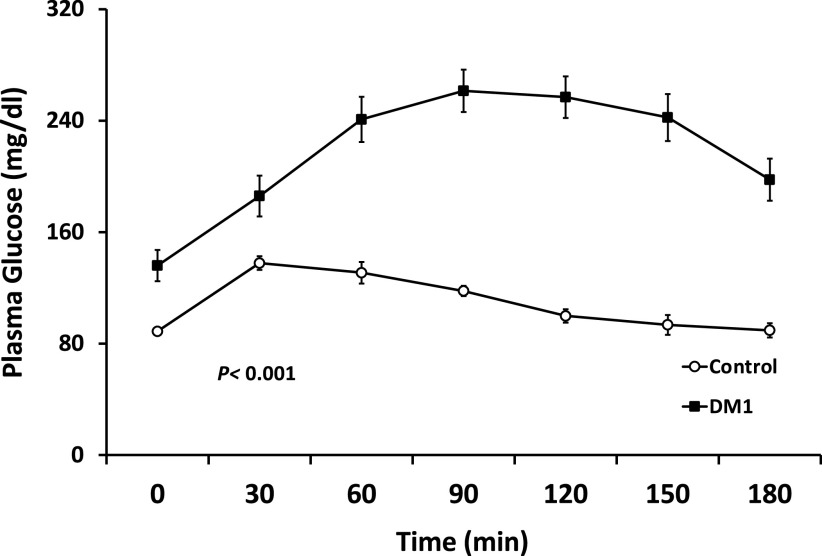
The time course for changes in plasma glucose in the two groups of adolescents. The *P* value indicates results of repeated measure ANOVA. DM1, type 1 diabetes.

**Figure 5.**
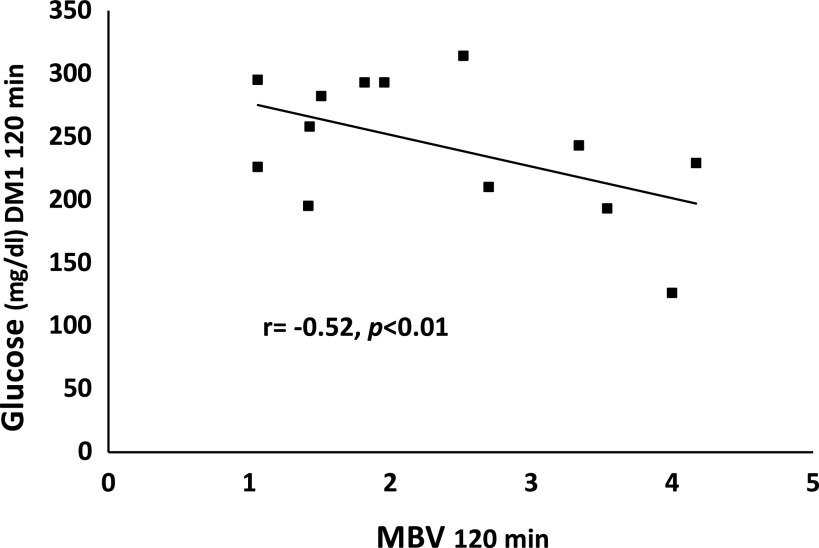
The negative correlation between plasma glucose and microvascular blood volume at 120 min after glucose ingestion. DM1, type 1 diabetes; MBV, microvascular blood volume.

Glucose was higher during the OGTT in the DM1 group versus control (Fig. 4), as was the glucose AUC (35,186 ± 2,091 vs. 19,784 ± 481 min/mg/dL, *P* < 0.001). Interestingly, there was an inverse correlation between MBV and glucose at 120 min (*r* = −0.52, *P* < 0.01, Fig. 5). Plasma insulin rose promptly after glucose ingestion in controls, more slowly and less markedly in the DM1 subjects (Table 2). Note, in six of the DM1 subjects, antibody interference prevented accurate measurement of insulin concentrations.

**Table 2.** Plasma insulin concentrations

	Controls	DM1
Time (min)	mU/L	mU/L
Baseline	6.7 ± 1.1	8.0 ± 4.0
30	78 ± 8	29 ± 13*
60	62 ± 10	20 ± 8*
90	51 ± 6	27 ± 10
120	31 ± 6	23 ± 8
150	21 ± 8	19 ± 8
180	18 ± 5	22 ± 9

*P < 0.01 vs. control.

### Role of Fitness and Body composition: V̇O_2max_, %BF, and BMI

There were no differences noted between control and DM1 subjects for any of these variables. In addition, there were no statistically significant correlations between these three variables and either basal- or post-OGTT measures of macro- or microvascular function.

## DISCUSSION

This study is the first to comprehensively assess the impact of DM1 on macro- and microvascular function using two indices of vascular stiffness (cfPWV and AI) and three measures of NO-dependent vascular relaxation (FMD, PIFV, and microvascular perfusion by CEU). Considering first the NO-dependent measures, at *time 0,* the postischemic vasodilatory response (FMD) was significantly greater (*P* < 0.02) in the control subjects, indicating an impaired conduit artery endothelial NO-dependent response in DM1 subjects. Interestingly, of those with impaired responses, FMD was similarly impaired in the six adolescents with DM1 of ≤3 yr duration (6.6 ± 1.1%) compared with the five children with ≥10 yr of DM1 (7.1 ± 1.7%), indicating that brachial artery endothelial dysfunction occurs early in the course of DM1. Our finding that 69% of participants with DM1 had impaired FMD responses is consistent with a prior report that found 60% of subjects with a >5-yr diabetes duration had impaired FMD (25), and 36% of children or adolescent participants with less than a 5-yr diabetes duration also had this finding (25, 26). The postischemic increment of brachial artery flow velocity trended higher in the control subjects (*P* = 0.06), suggesting that NO-dependent, postischemic resistance arteriolar vasodilation is impaired in DM1, consistent with endothelial dysfunction in small arterioles and larger arteries. For PIFV as well, the velocity increments were similarly low in those with diabetes for ≤3 yr and those with >9 yr history of DM.

We have previously shown that in the microvasculature of healthy subjects, insulin enhances capillary perfusion (27) and that insulin resistance can block that response (22) and even provoke a reduced perfusion response to insulin (28). Microvascular perfusion is enhanced by NO production (29), whereas diminished perfusion appears dependent on insulin-mediated endothelin-1 release (28). In response to the OGTT, microvascular perfusion (i.e., MBV) for participants with DM1 was unchanged from baseline to 60 min then declined significantly at 120 min compared with either the 0- or 60-min values (*P* < 0.01 for each), indicating a vasoconstrictive response by small arterioles and diminished perfusion of capillaries. The MBV in DM1 was also significantly less than the MBV of controls at 120 min, which was unchanged from baseline during the OGTT.

Several studies have reported increased vascular stiffness [measured as either AI (30) or cfPWV (31)] in DM1, including in adolescents and children (4, 5). In the large SEARCH study (*n* = 535 with DM1 and 241 controls), investigators assessed vascular stiffness using radial artery tonometry and cfPWV (5). The latter was not increased in DM1 compared with controls, but 9.9% of DM1 adolescents were considered to have abnormally rapid cfPWV. In this same study, average AI was modestly greater in the DM1 versus controls (2.1 ± 11 vs. −0.5 ± 11, *P* < 0.03). Given these finding in a large cohort, it is not surprising that we did not observe significant differences in either measure of vascular stiffness at baseline in the current study. However, looking beyond the baseline to the OGTT response, we found very clear between-group differences in augmentation index. Westerbacka and colleagues first pointed out that euglycemic-hyperinsulinemia in healthy individuals acutely lowers the augmentation index and this response was diminished or delayed in insulin-resistant adults with DM1 (30) and obesity (32, 33). Our current findings in adolescents with DM1 during insulin plus glucose administration are consistent with those prior findings. AI and cfPWV, each an index of vascular stiffness, behaved differently with the OGTT in our study population. This may be due to differing responses of the elastic aorta measured by cfPWV and the muscular brachial artery, which contributes to the AI measurement. Several previous studies have indicated that vasoactive agents can differentially affect cfPWV and AI, with the former (an elastic artery) correlating more strongly with changes in mean arterial pressure, whereas the latter responds to smooth muscle vasorelaxing stimuli (34, 35). Insulin, which stimulates NO release, may be playing that vasorelaxing role in the control children, but the effect is clearly absent in the children with DM1.

We also addressed what, if any, effect fitness had upon these measures of baseline or post-OGTT vascular stiffness and NO-mediated vasodilation. Some previous work suggested that adolescents and adults with DM1 may be less fit than peers (36) and this could impact vascular function (37). We note that this is not a uniform finding (38). Indeed, our subjects with DM1 had a slightly, but not significantly lower, mean V̇O_2max_ compared with controls, suggesting that fitness differences alone could not account for the altered NO-dependent vascular function we observed. Similarly, we did not find any differences in BMI, V̇O_2max_ or body fat composition that correlate with altered vascular function. We caution that both our adolescent control and DM1 subject groups were quite homogeneous with respect to these variables, which may have limited correlation analysis.

The OGTT response is impacted by both insulin secretion and resistance. In this study, compared with controls of similar age and weight, the OGTT AUC was significantly higher in the DM1 group (*P* < 0.001) despite exogenous insulin, suggesting either metabolic insulin resistance or mismatched insulin timing or dosage for a simple carbohydrate meal (or a combination of both). Insulin resistance and/or the higher plasma glucose concentrations may have contributed to the altered vascular responses observed in subjects with DM1. Russell et al. (39) reported that an oral glucose load decreased microvascular perfusion in healthy adults, though we did not see this in the healthy adolescents studied here. Interestingly, we recently found that in healthy adults, hyperglycemia (provoked by simultaneous intravenous glucose and octreotide infusion) acutely augments muscle microvascular perfusion (40). In contrast, both chronic insulin resistance [as seen with obesity (22), or metabolic syndrome (23)] and acute insulin resistance induced by raising plasma free fatty acid concentrations (41) provoke muscle microvascular vasoconstriction similar to that seen here in persons with DM1. Insulin resistance is commonplace in both adults and children with DM1 (42). This leads us to suspect that insulin resistance may be driving the aberrant response to OGTT observed in the current study.

Plasma incretin concentrations were not measured during this study but others have reported that GLP-1 and GIP responses to meal ingestion to be comparable in DM1 to healthy, age-matched controls (43). Inasmuch as GLP-1, like insulin, has microvascular vasodilatory actions in muscle (44, 45) we cannot exclude the possibility that in the current study a diminished response to incretins released following the OGTT contributed to the impaired vasodilation in the M1 adolescents. We note, however, that GLP-1 appears to retain its vasodilatory action in states of insulin resistance (45).

Although our study has significant strengths, there are several important limitations. Subcutaneous regular insulin was used in all participants with DM1 during the OGTT, this likely led to the lower insulin levels and more pronounced hyperglycemia following OGTT in the DM1 group, which could impact vascular responses. Furthermore, this study only correlated HbA1c values at the time of study, not early in diagnosis, although the HbA1c in the earlier period appears to better predict endothelial dysfunction (25).

### Conclusions

Here, we confirm that adolescents with DM1 have impaired baseline conduit artery NO-dependent vasodilation in response to shear stress and provide novel evidence of microvascular and large artery endothelial insulin resistance, consistent with impaired insulin action to stimulate NO-dependent vasodilation. These defects precede clinically evident macro-and microvascular disease or development of vascular stiffness in elastic or muscular arteries. Importantly, our findings reveal the presence of postprandial skeletal muscle microvascular insulin resistance in this population. More study is needed to determine whether episodes of diminished postprandial perfusion of muscle (or other tissues) occurs repetitively in adolescents or adults with DM1.

## ETHICAL APPROVAL

Written informed consent was obtained from all subjects. The study protocol and consent was approved by the University of Virginia Human Studies institutional review board.

## DATA AVAILABILITY

All data are available from the corresponding author on reasonable request.

## GRANTS

This work was supported by National Institutes of Health (NIH) Grants DK101944 and DK073059 (to E.J.B.) and in part by the National Center for Advancing Translational Sciences of the National Institutes of Health under Award Numbers KL2TR003016/ULTR003015 (to K.M.L. and W.B.H. as iTHRIV scholars). 

## DISCLAIMERS

The content is solely the responsibility of the authors and does not necessarily represent the official views of the National Institutes of Health.

## DISCLOSURES

No conflicts of interest, financial or otherwise, are declared by the authors.

## AUTHOR CONTRIBUTIONS

E.J.B. conceived and designed research; L.A.J., B.L., L.M.H., and E.J.B. performed experiments; L.A.J., B.L., K.M.L., W.B.H., N.Z.E., L.M.H., A.L.W., and E.J.B. analyzed data; B.L., W.B.H., A.L.W., and E.J.B. interpreted results of experiments; L.A.J., K.M.L., N.Z.E., and E.J.B. prepared figures; L.A.J., B.L., N.Z.E., and E.J.B. drafted manuscript; L.A.J., K.M.L., W.B.H., L.M.H., A.L.W., and E.J.B. edited and revised manuscript; L.A.J., B.L., K.M.L., W.B.H., N.Z.E., L.M.H., A.L.W., and E.J.B. approved final version of manuscript.
